# Prediction of Chemoresistance in Women Undergoing Neo-Adjuvant Chemotherapy for Locally Advanced Breast Cancer: Volumetric Analysis of First-Order Textural Features Extracted from Multiparametric MRI

**DOI:** 10.1155/2018/8329041

**Published:** 2018-05-03

**Authors:** M. M. Panzeri, C. Losio, A. Della Corte, E. Venturini, A. Ambrosi, P. Panizza, F. De Cobelli

**Affiliations:** ^1^Department of Radiology, Ospedale San Raffaele, Via Olgettina 60, 20132 Milan, Italy; ^2^Vita-Salute University, San Raffaele Scientific Institute, Milan, Italy

## Abstract

**Purpose:**

To assess correlations between volumetric first-order texture parameters on baseline MRI and pathological response after neoadjuvant chemotherapy (NAC) for locally advanced breast cancer (BC).

**Materials and Methods:**

69 patients with locally advanced BC candidate to neoadjuvant chemotherapy underwent MRI within 4 weeks from the start of therapeutic regimen. T2, DWI, and DCE sequences were analyzed and maps were generated for Apparent Diffusion Coefficient (ADC), T2 signal intensity, and the following dynamic parameters: *k*-trans, peak enhancement, area under curve (AUC), time to maximal enhancement (TME), wash-in rate, and washout rate. Volumetric analysis of these parameters was performed, yielding a histogram analysis including first-order texture kinetics (percentiles, maximum value, minimum value, range, standard deviation, mean, median, mode, skewness, and kurtosis). Finally, correlations between these values and response to NAC (evaluated on the surgical specimen according to RECIST 1.1 criteria) were assessed.

**Results:**

Out of 69 tumors, 33 (47.8%) achieved complete pathological response, 26 (37.7%) partial response, and 10 (14.5%) no response. Higher levels of AUCmax (*p* value = 0.0338), AUCrange (*p* value = 0.0311), and TME_75_ (*p* value = 0.0452) and lower levels of washout_10_ (*p* value = 0.0417), washout_20_ (*p* value = 0.0138), washout_25_ (*p* value = 0.0114), and washout_30_ (*p* value = 0.05) were predictive of noncomplete response.

**Conclusion:**

Histogram-derived texture analysis of MRI images allows finding quantitative parameters predictive of nonresponse to NAC in women affected by locally advanced BC.

## 1. Introduction

Neoadjuvant chemotherapy is the first-line treatment for locally advanced breast cancer and is a viable option to reduce the extent of surgery needed to achieve adequate resection [1], but tumor response may vary among different subtypes of cancers. On average, only 30% cancers respond completely to NAC [2]. Treating a chemoresistant BC with NAC may cause patient harm, because of the delay of surgery, with potential disease progression during NAC, and because of drug toxicity [3]. Magnetic resonance imaging (MRI), based on dynamic contrast enhanced (DCE) sequences and diffusion weighted imaging (DWI), is a well-established tool to assess pretreatment tumor extent and final response to NAC [4]. Indeed, multiparametric MRI is the most reliable currently available technique able to study morphology, vascularization, and cellular density. Parameters extracted from MR images are usually extracted from a 2D region of interest (ROI): the results are the average value obtained from an arbitrarily selected slice. Since breast cancer may have very heterogeneous composition (featuring the coexistence of necrotic areas, highly vascularized and cell-packed areas, fibrosis, and edema), nonunivocal results have been thus far been obtained in literature from MR parameters as predictive factors of response to NAC [3, 5]. More recent studies suggest that a volume-based histogram analysis of MRI parameters, including calculation of first-order texture kinetics, could allow a more accurate assessment of tumor heterogeneity intrinsic to breast cancer [6]. As a consequence, recent studies have been focusing at finding different texture metrics which could turn out useful in the characterization of malignant lesions and their response to neoadjuvant chemotherapy [7–9]. In our work, we wanted to evaluate the possible correlation between texture parameters and response to NAC, according to different histotypes in locally advanced BC.

## 2. Methods

### 2.1. Population

This retrospective study has been performed in accordance with the ethical standards as laid down in the 1964 Declaration of Helsinki and its later amendments.

Informed consent for the use of anonymized images and clinical data was obtained from all individual participants included in the study.

We reviewed the history of all patients with histopathologically proven (core-needle biopsy), locally advanced breast cancer undergoing neoadjuvant chemotherapy (NAC) between January 2012 and January 2016 at our institute. Apart from the histological verification of the invasive breast carcinoma, inclusion criteria comprised the presence of a measurable lesion, according to Response Evaluation Criteria in Solid Tumors (RECIST 1.1). The exclusion criteria were the presence of distant metastases. In patients presenting with more than one lesion, only the one with largest diameter was considered for study. All patients were managed according to multidisciplinary-based protocols in collaboration with in-house oncologists and surgeons. Baseline MRI evaluation was planned within 4 weeks before the beginning of the NAC protocol. All patients underwent surgical excision at our institute and final histopathological analysis was performed.

### 2.2. Neoadjuvant Chemotherapy

All patients received a treatment based on taxanes and anthracyclines. HER2 positive tumors also received Trastuzumab. NAC regimens included Adriamycin/Taxane + Cyclophosphamide/Methotrexate/Fluorouracil (ATCMF) for 15 patients, ATCMF + Herceptin (ATCMFH) for 8 patients, weekly Taxane/Herceptin + Fluorouracil/Epirubicin/Cyclophosphamide (WTH-FEC) for 9 patients, weekly Taxane/Adriamycin/Fluorouracil/Epirubicin/Cyclophosphamide (WAFEC) for 3 patients, Taxane/Herceptin/Pertuzumab (THP) for 2 patients, Fluorouracil/Epirubicin/Cyclophosphamide/Taxane/Herceptin (FECTH) for 4 patients, weekly Taxane/Fluorouracil/Epirubicin/Cyclophosphamide (WTFEC) for 14 patients, ATCM + Capecitabine (ATCMX) for 2 patients, Carboplatin/Taxane (CBDCAT) for 1 patient, and weekly Taxane (WT) for 1 patient. For 7 patients, the exact NAC regimens were not available.

### 2.3. MRI Examination Technique

All MRI studies were performed at our department on a 1,5T system (Achieva DStream, Philips Medical Systems) equipped with a dedicated double breast coil (Breast Sense Coil). Patients were in the prone position. Standard sequences were acquired: a T2-weighted Turbo Spin Echo (TSE) sequence was performed in the axial plane (FOV 270 × 331 mm^2^, TR 4100 ms, TE 120 ms, matrix 300 × 301, slice thickness 2.2 mm, gap 0.5 mm, and time of acquisition = 3′36′′). DWI was performed using a single-shot echo-planar image (EPI) sequence on the axial plane with the following parameters: FOV 280 × 336 mm^2^, TR/TE 7585/81 ms, matrix 156 × 132, slice thickness 3 mm, gap = 1 mm, and acquisition time 2′54′′. Sensitizing diffusion gradients were applied along the *x*-, *y*-, and *z*-axes with *b* value of 0 and 900 s/mm2.

A precontrast axial 3D T1-weighted Dixon Fast Field Echo (FFE) sequence (FOV 330 × 330 mm^2^, TR 12 ms, TE1 2.1 ms, TE2 4.0 ms, matrix 400 × 394, slice thickness 2.2 mm, gap = 0 mm, and time of acquisition = 1′30′′). Then, 0.1 mmol/kg of gadobutrol (Gadovist, Bayer) was injected intravenously (*v* = 2 ml/s), followed by 20 ml of saline, and 10 s later the T1-weighted sequence was repeated 5 times with the same parameters used in the precontrast acquisition. Following the acquisition, the axial precontrast T1-weighted sequence was subtracted from the axial postcontrast images.

### 2.4. Breast MRI Analysis

MRI studies were interpreted by two experienced radiologist; conventional images were available during interpretation. Tumor size was recorded as the longest dimension in the T1-weighted contrast-enhanced subtracted images on the pre-NAC MRI, following the Response Evaluation Criteria in Solid Tumor (RECIST). If the lesion was still present on the post-NAC MRI, its diameter was recorded too and the percent reduction in tumor size was then calculated. For patients with presence of multiple lesions, only the largest one was considered for analysis. Based on the percent reduction in tumor size and according to RECIST 1.1 criteria, the response to NAC was classified as complete response if the lesion had a reduction of 100%, partial response if the reduction was at least 30%, progression if the lesion increased of at least 20%, and stable disease if there was not partial response neither progression. Each lesion visualized on subtracted images was then categorized according to the MRI BI-RADS classification system (Breast Imaging Reporting and Data System, American College of Radiology).

### 2.5. Volumetric and Histogram Analysis

Diffusion weighted imaging and dynamic contrast enhanced and T2 sequences were imported using OLEA software (OLEA Sphere 3.0, OLEA Medical), which then generated maps for Apparent Diffusion Coefficient, T2 intensity projection, *k*-trans, area under curve (AUC), time to maximal enhancement (TME), wash-in rate, washout rate, and peak enhancement. VOIs were manually depicted slice by slice on the aforementioned maps. The software then generated histograms for each of the 8 VOIs analyzed. Finally, the first-order texture parameters were extrapolated from the histograms, including percentiles, maximum value, minimum value, range, standard deviation, mean, median, mode, skewness, and kurtosis.

### 2.6. Histopathological Analysis

Breast specimens, obtained before the pre-NAC MRI, were analyzed by an experienced pathologist. Tumor size and location were described. The histological type of breast cancer was defined according to the WHO classification. Tumor histopathological grade was assessed by the Elston–Ellis System. The assessment of estrogen receptor (ER) and progesterone receptor (PR) status was carried out by using the appropriate monoclonal antibody. Human epidermal growth factor receptor 2 (HER2) status was evaluated by both FISH and IHC. The Mib-1 monoclonal antibody was used to assess the Ki-67 status of specimen. Since breast cancer molecular subtypes defined by gene expression analysis have been shown to be reasonably approximated using classical IHC markers, breast lesions were classified according to their ER, PR, HER2, and Ki67 status: Luminal A lesions were ER positive, PR positive, and Ki67 < 14% and HER2 negative; Luminal B were ER positive and PR positive with either Ki67 > 14% or HER2 positive; HER2 enriched were ER negative, PR negative, and HER2 positive; triple-negative were ER, PR, and HER2 negative. Surgical specimens were analyzed to define the pathologic complete response (pCR) as the absence of microscopic residual invasive cancers. A pathological incomplete response was defined as the presence of microscopic invasive tumor in the final pathology. The presence of tumor positive lymph nodes was not considered in the assessment of response.

### 2.7. Statistical Analysis

For the analysis of correlation between texture parameters and response, quantitative variables were described by their average value and standard deviation, qualitative variables by absolute, and relative frequencies. For both complete responders (CR) and nonresponders (NR), the overall discrimination performance of each variable was evaluated through ROC analysis and the associated area under curve (AUC). Then, for CR and NR groups separately, the quantitative variables were dichotomized according to the top-left corner rule. Based on the ROC curve, an optimal cut-off was fixed, maximizing the quantity (1 − sensitivity)^2^  +  (1 − specificity)^2^. The performance of the cut-off at discriminating the group of patients under consideration from the others was measured by sensitivity, specificity, positive predictive value, negative predictive value, and accuracy. All *p* values were computed by means of permutation methods to avoid and distributional assumption or asymptotic approximation. *p* values < 0.05 were considered significant. All the analysis was performed in R environment.

## 3. Results

### 3.1. Demographics

We enrolled 69 patients with histopathologically proven breast cancer. Mean age was 47.9 years, with a range between 29 and 80 years. Out of 69 patients under examination, 33 (47.8%) achieved complete pathological response, 26 (37.7%) achieved a partial response, and 10 (14.5%) did not respond to treatment. The mean tumor size was 3.8 cm (range, 1.1–10.0 cm). The axillary lymph nodes were positive in 44 cases and negative in 25 cases. Histological grades of the tumors were grade 1 or 2 in 27 cases and 3 in 42 cases. Regarding distribution of the histological subtypes, 6 (8.7%) were Luminal A, 24 (34.8%) were Luminal B, 21 (30.4%) were triple negative, and 18 (26.1%) were Her2-enriched.

### 3.2. Correlations between Texture Parameters and Pathological Response

No significant correlations were found between histogram-derived variables and complete responders. Univariate analysis of the correlations between texture parameters and the nonresponder category yielded significant results for the variables reported in the table (Table 1). AUC_MAX_ and AUC_RANGE_ were found to be higher in the NR group (threshold levels of 536,645.5 (*p* value = 0.0338) and 534,858.3 *p* value = 0.0311, resp.); TME_75_ was also higher in the NR group (threshold: 373 *p* value = 0.0452), whereas lower values of washout_10_ (*p* value = 0.0417), washout_20_ (*p* value = 0.0138), and washout_25_ (*p* value = 0.0114) were correlated to the NR group. No significant correlations between mean values of any of these variables and nonresponder group were found.

**Figure 1:**
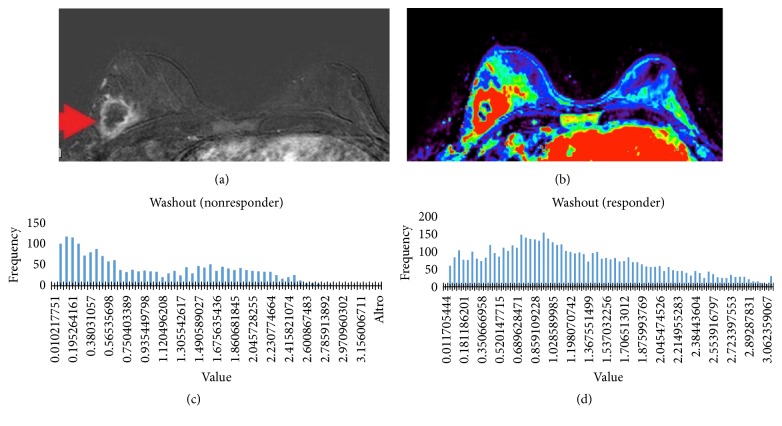
Appearance of a lesion on both subtraction (a) images and washout maps (b). Histogram of washout value in a responder patient (c) and in a nonresponder patient (d).

**Figure 2:**
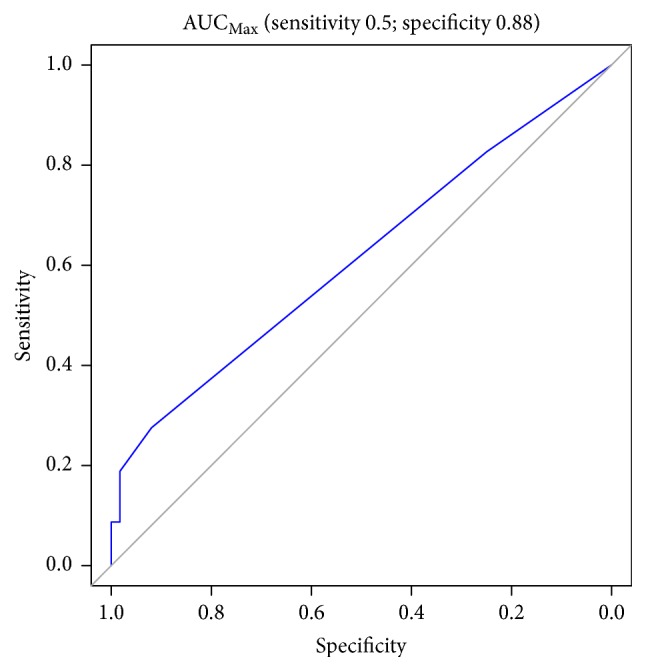
ROC curve of AUCmax for prediction of pCR.

**Figure 3:**
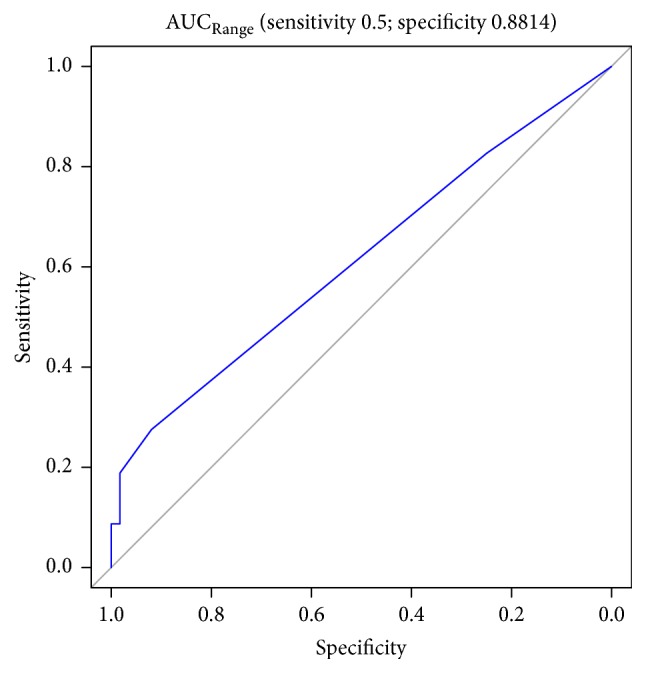
ROC curve AUC range for prediction of pCR.

**Figure 4:**
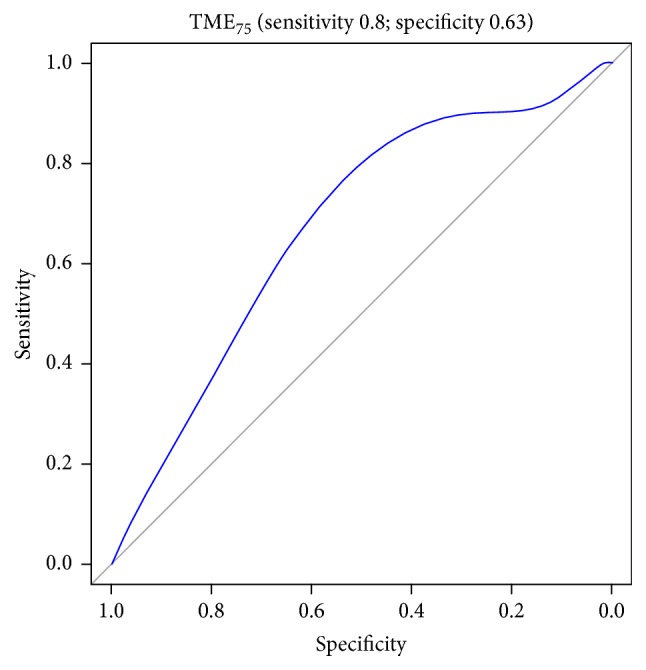
ROC curve of TME_75_ for prediction of pCR.

**Figure 5:**
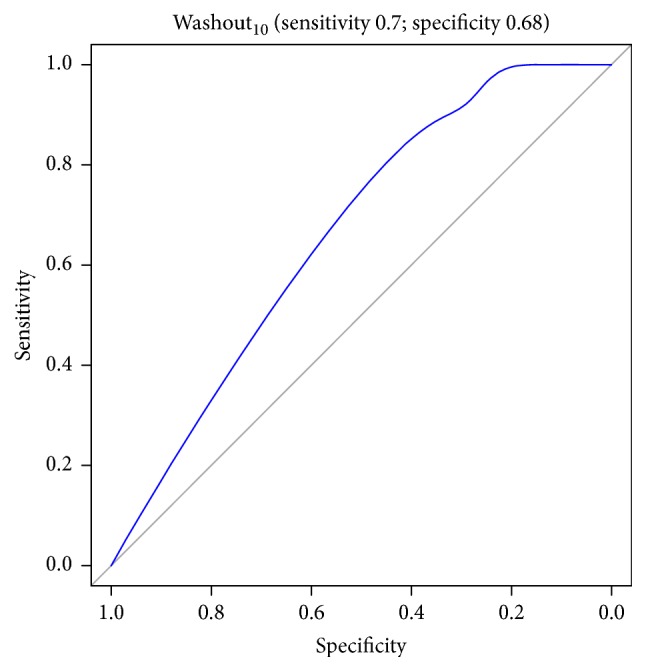
ROC curve of washout_10_ for prediction of pCR.

**Figure 6:**
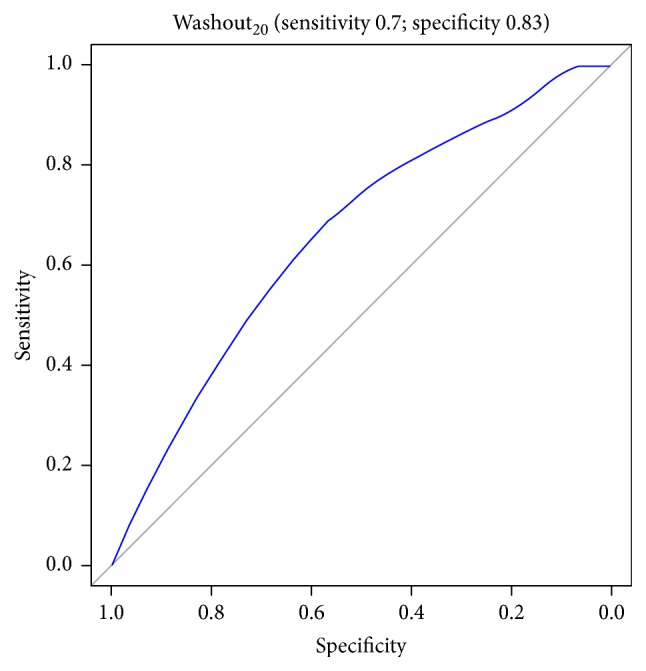
ROC curve of washout_20_ for prediction of pCR.

**Figure 7:**
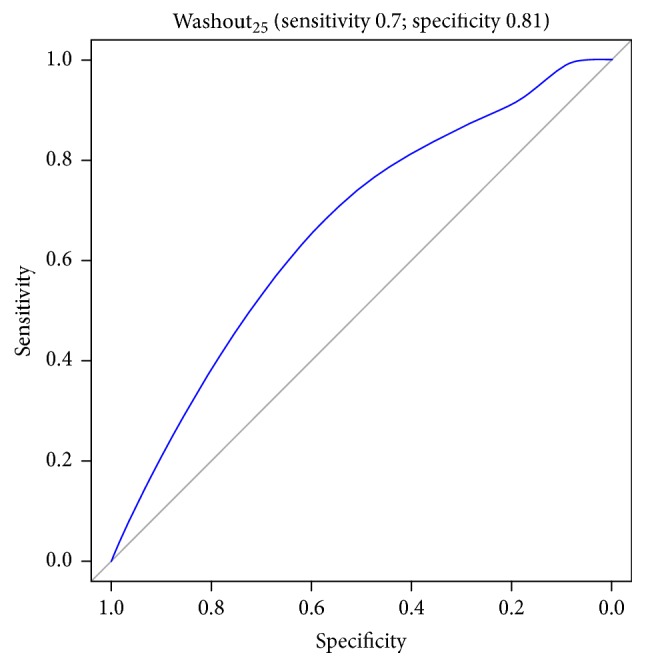
ROC curve of washout_25_ for prediction of pCR.

Figures 1–7 show the ROC curve of AUCmax, AUC range, TME_75_, washout_10_, washout_20_, and washout_25_ for prediction of pCR. The area under the ROC curve (AUC) of the AUCmax was 0.712 (*p* < 0.034), and its cut-off value was higher than 536.645 (sensitivity = 50%; specificity = 88.1%). The AUC of the AUC range was 0.715 (*p* < 0.031), and its cut-off value was higher than 534.858 (sensitivity = 50%; specificity = 88.1%). The AUC of the TME_75_ was 0.7 (*p* < 0.045), and its cut-off value was higher than 373.035 (sensitivity = 80%; specificity = 62.7%). The AUC of the washout_10_ was 0.703 (*p* < 0.042), and its cut-off value was higher than 0.006 (sensitivity = 70%; specificity = 67.8%). The AUC of the washout_20_ was 0.746 (*p* < 0.014), and its cut-off value was higher than 0.011 (sensitivity = 70%; specificity = 83.1%). The AUC of the washout_25_ was 0.753 (*p* < 0.011), and its cut-off value was higher than 0.029 (sensitivity = 70%; specificity = 81.4%).

## 4. Discussion

The use of neoadjuvant chemotherapy (NAC) has become the standard treatment of locally advanced breast cancer: previous studies have widely demonstrated that a pathological complete response (pCR) after NAC correlates with a higher disease-free survival. In nonresponsive patients, surgery should be carried out as soon as possible to avoid the harm of a disease progression [3, 4]. Several studies already investigated the predictive markers of response to NAC, with contradictory results [4, 5, 7]. More recent works suggest that a volume-based histogram analysis of MRI parameters, including calculation of first-order texture kinetics, could allow a more accurate assessment of tumor heterogeneity intrinsic to breast cancer [6, 9]. In our study, we evaluated both textural and qualitative features of tumors (including histopathological and MRI morphological data) as predictive factors for response to NAC.

Univariate analysis between texture parameters and pathological response yielded no significant results for prediction of complete response. AUC_MAX_ and AUC_RANGE_ were shown to be significantly higher in NR to NAC (*p* < 0.05). AUC stands for area under curve of enhancement, and it gives a measure of how much contrast is uptaken by the lesion. AUC_MAX_ may reflect the presence of highly hypervascular voxels in the most malignant part of the tumor which will not respond to treatment. In literature, no studies assess the relevance of AUC as a predictive factor for pathological response, but in a recent study by Pickles et al., histogram-derived AUC values (AUC_25_, AUC_90_, and AUC_95_) were shown to be a prognostically unfavourable factor for both DFS and OS [7]. On the other hand, AUC_RANGE_ may be a reflection of the difference between the most vascularized and the least vascularized voxel within the lesion. It is possible to speculate that high values of AUC_RANGE_ in cancers not responding to neoadjuvant treatment might be due to the presence of both hypervascular, highly malignant voxels, and less vascularized parts of the tumor, such as, for example, necrotic parts peculiar of aggressive cancers or fibrotic areas in the less aggressive subtypes (Luminal A). Interestingly, in our study mean and median AUC did not yield significant results; therefore, only through histogram analysis we were able to demonstrate the relevance of AUC of enhancement in the prediction of breast cancer response to neoadjuvant chemotherapy. Time to maximal enhancement (i.e., time between injection of contrast and peak of enhancement.) 75th percentile was also found to be higher in NR than CR. Data on the literature about the significance of time to maximal enhancement are limited. However, we may speculate that it takes longer for the contrast to get to its peak because of poorer efficacy of vascularization in cancers which will not respond to neoadjuvant chemotherapy. Indeed, in this sense, a less “avid” tumoral bed would lead to less efficient drug delivery. In our studies, lower values of washout_10_, washout_20_, and washout_25_ were all predictors of NR to NAC. Washout rate is a measure of the flow back to plasma after being diffused into interstitium. In literature, this finding is apparently in contrast with the recent study by Wu et al. 2016, which showed that heterogeneity of the tumor subregion showing higher values of washout rate predicted response to NAC [10]. Furthermore, Chaudhury et al. recently showed that heterogeneity in intratumoral regions with rapid gadolinium washout was associated with estrogen receptor status and nodal metastasis [11]. However, it must be noted that lower values of washout rate in chemoresistant cancers is only occurring at low percentiles. The possible explanation for this is that necrotic areas showing low washout values may be predominating at lower percentiles in highly heterogeneous cancers. Mean and median values of washout rate did not yield significant results: this is in accordance with Abramson et al. 2013, who studied, among other variables, mean washout in a cohort of patients undergoing NAC but did not find significant results in the predictive setting [12].

Regarding diffusion weighted imaging, no significant correlations were found between ADC texture metrics and response to chemotherapy in our study. While literature has shown that in some solid tumors lower ADC values are associated with high cell density and better response to chemotherapy [13], there are still controversial data concerning ADC as a predictive factor for therapeutic response in breast cancer [3].

Our study has some limits: different chemotherapy regimens were used according to multidisciplinary-based protocols and may have affected the response rate. Furthermore, analysis of DCE parameters was estimated from semiquantitative data of conventional dynamic study with a low temporal resolution (8′30′′ for whole dynamic study, 1′30′′ for each acquisition); data extracted from a perfusional dynamic sequence would be more reliable, but at the expense of lower spatial resolution actually not acceptable in clinical breast MRI.

Histogram-derived texture analysis of MRI images allows finding quantitative parameters predictive of nonresponse to NAC, in women affected by locally advanced breast cancer. Nonresponsiveness could also be associated with some morphological and histopathological features. The clinical implication of this method is to reconsider the treatment planning in patients who will likely not benefit from NAC, sparing the toxicity of ineffective treatment and avoiding delay in surgical treatment. These findings stress the importance of texture analysis for the assessment of tumor heterogeneity, with the goal of investigating future correlations with histopathological prognostic factors.

## Figures and Tables

**Table 1:** Univariate analysis of the correlations between texture parameters and the nonresponder category. Negative predictive value: NPV, positive predictive value: PPV, accuracy: ACC, and area under the curve: AUC.

Parameter	Threshold	Sensitivity	Specificity	PPV	NPV	ACC	AUC	*p* value
AUC_MAX_	536645,51	0,50	0,88	0,42	0,91	0,83	0,71	0,03
AUC_RANGE_	534858,33	0,50	0,88	0,42	0,91	0,83	0,72	0,03
TME_75_	373,03	0,80	0,63	0,27	0,95	0,65	0,70	0,05
Washout_10_	0,01	0,70	0,68	0,27	0,93	0,68	0,70	0,04
Washout_20_	0,01	0,70	0,83	0,41	0,94	0,81	0,75	0,01
Washout_25_	0,03	0,70	0,81	0,39	0,94	0,80	0,75	0,01
